# The Bradycardic Agent Ivabradine Acts as an Atypical Inhibitor of Voltage-Gated Sodium Channels

**DOI:** 10.3389/fphar.2022.809802

**Published:** 2022-05-02

**Authors:** Benjamin Hackl, Peter Lukacs, Janine Ebner, Krisztina Pesti, Nicholas Haechl, Mátyás C Földi, Elena Lilliu, Klaus Schicker, Helmut Kubista, Anna Stary-Weinzinger, Karlheinz Hilber, Arpad Mike, Hannes Todt, Xaver Koenig

**Affiliations:** ^1^ Department of Neurophysiology and Neuropharmacology, Medical University of Vienna, Vienna, Austria; ^2^ ELKH, Plant Protection Institute, Centre for Agricultural Research, Martonvásár, Hungary; ^3^ Department of Biochemistry, ELTE Eötvös Loránd University, Budapest, Hungary; ^4^ Semmelweis University, School of Ph.D. Studies, Budapest, Hungary; ^5^ Department of Pharmacology and Toxicology, University of Vienna, Vienna, Austria

**Keywords:** ivabradine, S16257, voltage-gated sodium channel, conduction cell, ventricular cardiomyocyte, atypical inhibitor

## Abstract

**Background and purpose:** Ivabradine is clinically administered to lower the heart rate, proposedly by inhibiting hyperpolarization-activated cyclic nucleotide-gated cation channels in the sinoatrial node. Recent evidence suggests that voltage-gated sodium channels (VGSC) are inhibited within the same concentration range. VGSCs are expressed within the sinoatrial node and throughout the conduction system of the heart. A block of these channels thus likely contributes to the established and newly raised clinical indications of ivabradine. We, therefore, investigated the pharmacological action of ivabradine on VGSCs in sufficient detail in order to gain a better understanding of the pro- and anti-arrhythmic effects associated with the administration of this drug.

**Experimental Approach:** Ivabradine was tested on VGSCs in native cardiomyocytes isolated from mouse ventricles and the His-Purkinje system and on human Na_v_1.5 in a heterologous expression system. We investigated the mechanism of channel inhibition by determining its voltage-, frequency-, state-, and temperature-dependence, complemented by a molecular drug docking to the recent Na_v_1.5 cryoEM structure. Automated patch-clamp experiments were used to investigate ivabradine-mediated changes in Na_v_1.5 inactivation parameters and inhibition of different VGSC isoforms.

**Key results:** Ivabradine inhibited VGSCs in a voltage- and frequency-dependent manner, but did not alter voltage-dependence of activation and fast inactivation, nor recovery from fast inactivation. Cardiac (Na_v_1.5), neuronal (Na_v_1.2), and skeletal muscle (Na_v_1.4) VGSC isoforms were inhibited by ivabradine within the same concentration range, as were sodium currents in native cardiomyocytes isolated from the ventricles and the His-Purkinje system. Molecular drug docking suggested an interaction of ivabradine with the classical local anesthetic binding site.

**Conclusion and Implications:** Ivabradine acts as an atypical inhibitor of VGSCs. Inhibition of VGSCs likely contributes to the heart rate lowering effect of ivabradine, in particular at higher stimulation frequencies and depolarized membrane potentials, and to the observed slowing of intra-cardiac conduction. Inhibition of VGSCs in native cardiomyocytes and across channel isoforms may provide a potential basis for the anti-arrhythmic potential as observed upon administration of ivabradine.

## Introduction

Ivabradine is clinically approved for the treatment of stable angina pectoris and heart failure (Rushworth et al., 2011; Scicchitano et al., 2014). The drug’s bradycardic effect is commonly believed to rely on the selective inhibition of hyperpolarization-activated cyclic nucleotide-gated (HCN) channels (Thollon et al., 1994; Bois et al., 1996; Bucchi et al., 2002; DiFrancesco and Camm, 2004; Bucchi et al., 2006), which mediate the “funny” pacemaker current I_f_ in the sinoatrial node (SAN).

The view of selective HCN channel blockade has been questioned recently by new *in vitro* findings demonstrating an inhibition of ERG potassium (Lees-Miller et al., 2015; Melgari et al., 2015; Haechl et al., 2019) and voltage-gated sodium channels [VGSCs; (Haechl et al., 2019)]. Inhibition of VGSCs by ivabradine explains the reduced maximal upstroke velocity of the cardiac action potential (AP) in dog Purkinje fibers and guinea pig papillary muscle (Pérez et al., 1995; Koncz et al., 2011), and is likely to be involved in the observed rate-dependent prolongation of the atrio-His (AH) interval in anesthetized pigs (Verrier et al., 2014; Verrier et al., 2015) and decreased conduction velocity in the atrioventricular node (AVN) and ventricles of mice *in vivo* (Amstetter et al., 2021).

Ivabradine free plasma concentrations are in the nM range after standard dosing but reported half inhibitory concentrations (IC_50_) for the inhibition of HCN, hERG, and VGSCs, which lie in the µM range, suggest a necessary tissue accumulation of the lipophilic drug to cause the aforementioned physiological effects.

Na_v_1.5 is the predominant VGSC isoform expressed in the myocardium (Zimmer et al., 2014); small contributions are ascribed to skeletal (Na_v_1.4) and neuronal (Na_v_1.1-1.3, Na_v_1.6-Na_v_1.9) channel isoforms (Zimmer et al., 2014). VGSCs mediate the upstroke of the AP in atrial and ventricular cardiomyocytes and contribute to impulse conduction in the SAN (Benson et al., 2003; Maier et al., 2003; Lei et al., 2004; Lei et al., 2008; Li et al., 2020) and the cardiac conduction system (Schott et al., 1999; Lei et al., 2008). Consequently, mutations within the SCN5A gene encoding for Na_v_1.5 have been linked to the sick sinus syndrome (Benson et al., 2003; Lei et al., 2008), cardiac conduction defects (Schott et al., 1999; Lei et al., 2008), and different forms of arrhythmias (Bezzina et al., 1999; Darbar et al., 2008).

A blockade of Na_v_1.5 per se can exert anti- or pro-arrhythmic activity (Tamargo et al., 2015). Recent meta-analyses suggested an increased risk of atrial fibrillation associated with ivabradine treatment (Martin et al., 2014; Tanboğa et al., 2016; Mengesha et al., 2017). On the other hand, ivabradine was able to control ventricular arrhythmias in catecholaminergic polymorphic ventricular tachycardia (Vaksmann and Klug, 2018; Kohli et al., 2020) and junctional ectopic tachycardia (Al-Ghamdi et al., 2013; Dieks et al., 2016; Kumar et al., 2017; Ergul et al., 2018; Ergul and Ozturk, 2018; Mert et al., 2018; Janson et al., 2019; Krishna et al., 2019; Kumar et al., 2019). Recently, ivabradine has also been considered as a rate control therapy for atrial fibrillation (Moubarak et al., 2014; Kosiuk et al., 2015; Turley et al., 2016; Wongcharoen et al., 2016; Fossati et al., 2017), with a current clinical trial to test for this potential new indication (Fontenla et al., 2019), and for other forms of atrial and ventricular tachyarrhythmias [e.g., (Cohen et al., 2020; Kohli et al., 2020)]. Moreover, ivabradine is currently being investigated for its autochthone and cardioprotective actions (Heusch, 2008; Kleinbongard et al., 2015; Heusch and Kleinbongard, 2016), for post-infarction (Suffredini et al., 2012) and post-heart transplantation treatment (Rivinius et al., 2020), and for its anti-epileptic potential (Cavalcante et al., 2019; Iacone et al., 2021).

Given the diversity of potential future off-label indications for ivabradine, the goal of the present study was to investigate the pharmacological action of ivabradine on VGSCs in sufficient detail in order to gain a better understanding of the pro- and anti-arrhythmic effects associated with the administration of this drug.

## Methods

### Isolation of Cardiomyocytes

Cardiomyocytes were isolated using a Langendorff preparation as previously described (Koenig et al., 2011). Briefly, mice aged 15–25 weeks were killed by cervical dislocation, and ice-cold Ca^2+^-free solution was injected into the ventricles to stop contractions and rinse free of blood. Hearts were rapidly excised and a cannula was inserted into the aorta. The heart was then mounted onto a Langendorff setup and retrograde perfusion was started with a Ca^2+^-free solution (in mM: 134 NaCl, 11 glucose, 4 KCl, 1.2 MgSO4, 1.2 Na2HPO4, 10 HEPES, pH was adjusted to 7.35 with NaOH) until the solution became clear (about 1min). The solution was then changed to the same Ca^2+^-free solution additionally containing 0.17 mg/ml LiberaseTH (Roche) and perfusion was maintained for 18 min at 37°C to allow for enzymatic digestion. During the entire procedure 10 mM of 2,3-butanedione monoxime (BDM, Sigma) was added to inhibit the contraction of cardiomyocytes and increase their viability. Thereafter, atria were removed and ventricles were cut into crude pieces. The tissue solution mix was incubated on a shaker water bath at 37°C, and calcium concentration was subsequently increased to 200 µM over a period of one hour in a total of five dilution steps. Pieces of digested ventricular tissue were triturated to liberate cardiomyocytes. After centrifugation (3 min, 500 rpm), the cells were resuspended in Minimum Essential Medium (MEM) alpha (Gibco), containing ITS media supplement (Sigma) diluted 1:100 (final concentration of 10 mg/ml insulin, 5.5 mg/ml transferrin, and 5 ng/ml selenite), 4 mM L-glutamine, 50 u/ml penicillin, 50 mg/ml streptomycin, and 25 mM blebbistatin (Sigma). The cells were plated on Matrigel (Becton Dickinson)-coated culture dishes.

Single Purkinje fibers (PF) were isolated as previously described (Ebner et al., 2020) by a procedure identical to the isolation of ventricular cardiomyocytes, except for additional enzyme incubation and additional trituration steps. After retrograde perfusion of the heart, the ventricles were cut open and placed in a petri dish containing 0.17 mg/ml LiberaseTH (Roche) dissolved in perfusion buffer. This additional incubation time for 8 min at room temperature further liberates PF and results in a better overall PF yield. The vast majority of isolated cells after the additional enzyme incubation step are ventricular cardiomyocytes, with a 1–10% fraction of PF. To identify PF, we used a transgenic mouse line (Cx40 eGFP/+; C57BL10 background) expressing enhanced GFP (eGFP) under the control of the connexin 40 (Cx40) gene promotor (Miquerol et al., 2004). Cx40 is a marker of the cardiac conduction system with strong expression in the atria, the AV-node, and the His-Purkinje system (Miquerol et al., 2004) but absent in the ventricles. During the isolation procedure, the atria are removed so that no eGFP positive atrial cardiomyocytes are collected. Hence, the eGFP signal allowed us to unambiguously identify PF in our preparation.

### Cell Culture and Transfection

For manual patch-clamp experiments, tsA-201 cells (American Type Culture Collection, Manassas, VA) were cultured in Dulbecco’s Modified Eagle Medium (Invitrogen, Vienna, Austria) supplemented with 10% fetal bovine serum, and incubated at 37°C in a humidified incubator with 5% CO_2_. Na_v_1.5 channels were expressed by transfecting tsA-201 cells with 0.5–1.5 µg of plasmid DNA per 3.5 cm culture dish. Human Na_v_1.5 was cloned (hH1; (Gellens et al., 1992)) and the respective sequence was inserted into a pEGFP-N2 vector (Clonetech) (Zimmer et al., 2002). The cells were transfected using Polyethyleneimine (PEI; Polysciences, Inc., Cat. No: 23966) following a custom-made protocol. The plasmid DNA was diluted in 80 µL 150 mM NaCl and vortexed briefly. Thereafter, 20 µL of PEI (10 µM in H2O) was added and vortexed again. The mixture was allowed to rest for 15 min and was then added to a 3.5 cm culture dish containing tsA-201 cells in a growth medium.

For automated patch-clamp experiments, recombinant rNa_v_1.2, rNa_v_1.4, and rNa_v_1.5 channel-expressing stable cell lines were generated as described before (Lukacs et al., 2018; Földi et al., 2021) by transfection of rNa_v_1.x BAC DNA constructs into HEK 293 cells (ATCC CRL-1573) using FuGENE HD (Promega, Fitchburg, WI) transfection reagent according to the manufacturer’s recommendations. The cell clones with stable vector DNA integration were selected by the addition of Geneticin (Life Technologies, Carlsbad, CA) antibiotic to the culture media (400 mg/ml) for 14 days. HEK293 cells were maintained in Dulbecco’s Modified Eagle Medium; high glucose was supplemented with 10% v/v fetal bovine serum, 100 U/ml of penicillin/streptomycin, and 0.4 mg/ml of Geneticin (Life Technologies, Carlsbad, CA). For experiments, the cells were plated onto T75 flasks, and cultured for 24–36 h. Before automated electrophysiology experiments, the cells were dissociated from the dish with accutase (Corning), shaken in a serum-free medium for 30 min at room temperature, then centrifuged, and resuspended into the extracellular solution at a concentration of 5 × 10^6^ cells/mL.

### Manual Patch-Clamp Technique

Sodium current through human Na_v_1.5 channels heterologously expressed in tsA-201 cells were recorded 48 h after transfection The pipette solution contained 105 mM CsF, 10 mM NaCl, 10 mM EGTA, and 10 mM HEPES, pH = 7.3 adjusted with CsOH. The external bathing solution consisted of (in mM): 140 NaCl, 2.5 KCl, 1 CaCl2, 1 MgCl2, and 10 HEPES; pH = 7.4 adjusted with NaOH. For recordings from ventricular cardiomyocytes and PF sodium concentration in the bath solution was reduced with equimolar replacement by NMDG, in mM: 15 NaCl, 125 NMDG, 2.5 KCl, 1 CaCl2, 1 MgCl2, and 10 HEPES, pH 7.3 with CsOH. A calculated liquid junction potential of −13.7 mV (Clampex 10.2) was not corrected for. Currents were recorded in the whole-cell mode of the patch-clamp technique using the voltage-clamp mode. Recordings were performed at room temperature (22 ± 2°C) using an Axoclamp 200B or 700B patch-clamp amplifier (Axon Instruments, Union City, CA). Pipettes were formed from aluminosilicate glass (A120-77-10; Science Products, Hofheim, Germany) with a P-97 horizontal puller (Sutter Instruments, Novato, CA) and had resistances between 1 and 2 MΩ when filled with the respective pipette solution, and between 2 and 4 MΩ when in the whole-cell configuration. Series resistance was not compensated. Data acquisition was performed with pClamp 11.0 software (Axon Instruments) through a 16-bit A-D/D-A interface (Digidata 1440 or 1550; Axon Instruments). Data were analyzed with Clampfit 10.2 (Axon Instruments) and Prism 5.01 (GraphPad Software, San Diego, CA) software. Rapid solution exchange was performed using a DAD-8-VC superfusion system (ALA Scientific Instruments, Westbury, NY).

### Automated Patch-Clamp Technique

Ensemble voltage-clamp recordings were performed on an IonFlux Mercury instrument (Fluxion Biosciences). Cell suspension, intracellular solution, and drug-containing extracellular solution were pipetted into 384-well IonFlux microfluidic ensemble plates. The composition of the solutions (in mM) was as follows: Intracellular solution: 50 CsCl, 10 NaCl, 60 CsF, 20 EGTA, 10 HEPES and pH is 7.2 (adjusted with 1 M CsOH). Extracellular solution: 140 NaCl, 4 KCl, 1 MgCl_2_, 2 CaCl_2_, 5 D-Glucose, and 10 HEPES; pH is 7.4 (adjusted with 1 M NaOH). The osmolality of intra- and extracellular solutions was set to ∼320 and ∼330 mOsm, respectively. Data were sampled at 20 kHz, and filtered at 10 kHz. The experiments were carried out at room temperature. The holding potential was set to −150 mV to minimize the voltage-dependent rundown of the current and to ensure that all channels are in the resting state. We used a voltage-clamp protocol that was optimized for high information content and high temporal resolution (Lukacs et al., 2021; Pesti et al., 2021). It consisted of 17 depolarizing pulses (Figure 3A). The whole sequence was repeated every second throughout the time course of the experiment and allowed us to continuously assess the steady-state inactivation (SSI), recovery from inactivation (RFI), and state-dependent onset (SDO) (Lukacs et al., 2021). It thus enabled us to continuously monitor the changes in the steady-state availability curve, the kinetics of recovery from inactivation, and the kinetics of state-dependent onset at 1-s resolution. For a detailed description and explanation of the protocol, and the process of automated patch-clamp data analysis, please see (Lukacs et al., 2021). At every second, steady-state availability vs. holding potential, recovery from inactivation vs. time, and state-dependent onset plots were automatically constructed and fitted. The steady-state availability curves were fit using the Boltzmann function: I = I_max_/{1 + exp[(V_p_−V_1/2_)/k]}, where V_p_ is the pre-pulse potential, V_1/2_ is the voltage where the curve reached its midpoint, and k is the slope factor. Recovery from inactivation was fitted with a double-exponential function I = *Σ*_i=1,2_ A_i_ * [1−exp(−t_p_/τ_i_)], where A_i_ is the amplitude, τ_i_ is the time constant of recovery, and t_p_ is the duration of the interpulse interval. The second time constant was always constrained to 150 ms. Conductance-voltage curves were fit using a Boltzmann function: G/Gmax = 1/{1 + exp[(V_1/2_ - V)/k]}

### Ivabradine

Ivabradine was purchased from Sigma Aldrich (SML0281), dissolved in Milli-Q water at a stock concentration of 100 mM, and stored in aliquots at -20°C. All solutions were prepared freshly on the day of the experiment by diluting the ivabradine stock to the respective concentrations as given within this study. The chemical properties of ivabradine were calculated using the chemicalize.com website (ChemAxon, Budapest, Hungary).

### Molecular Drug Docking

All cavity-lining residues between rNa_v_1.5 (cryo-EM structure) and hNa_v_1.5 are identical, and thus docking was performed with the cryo-EM structure (PDB code: 6UZ0, resolution 3.24 Å). Ivabradine in the protonated form (pK_a_ = 9.4), was docked into the 6UZO structure, using the program Gold 2020.1 (Cambridge Data Centre, Cambridge, United Kingdom, RRID: SCR_000188) (Jones et al., 1997). The binding site radius was set to 10 Å around the geometric center of flecainide. A total of 100,000 operations of the GOLD genetic algorithm with the “ChemScore” fitness scoring function were used to dock the compound, with the 20 highest ranked poses analyzed in detail. The visualization of results was done with PyMol (RRID:SCR_000305) 1.7.2 (Schrödinger, L. L. C. (2017). The PyMOL molecular graphics system was used, Version 1.8. 2015). A more detailed description of drug docking can be found in the Supplementary Material S1.

### Statistics

Data are given as mean ± SEM throughout the study. Statistical differences of data derived under two conditions, e.g., control versus ivabradine, were tested with a two-sided Student’s t-test when data were normally distributed, and with a Mann-Whitney test when data were not normally distributed. In case data were derived from the very same cell, a respective paired test was used. When more than two groups were compared, ANOVA with Tukey’s post-hoc test was used. A *p*-value of <0.05 was considered to indicate statistical significance.

## Results

### Ivabradine Does Not Affect the Voltage-Dependence of Na_v_1.5 Activation

**FIGURE 1:**
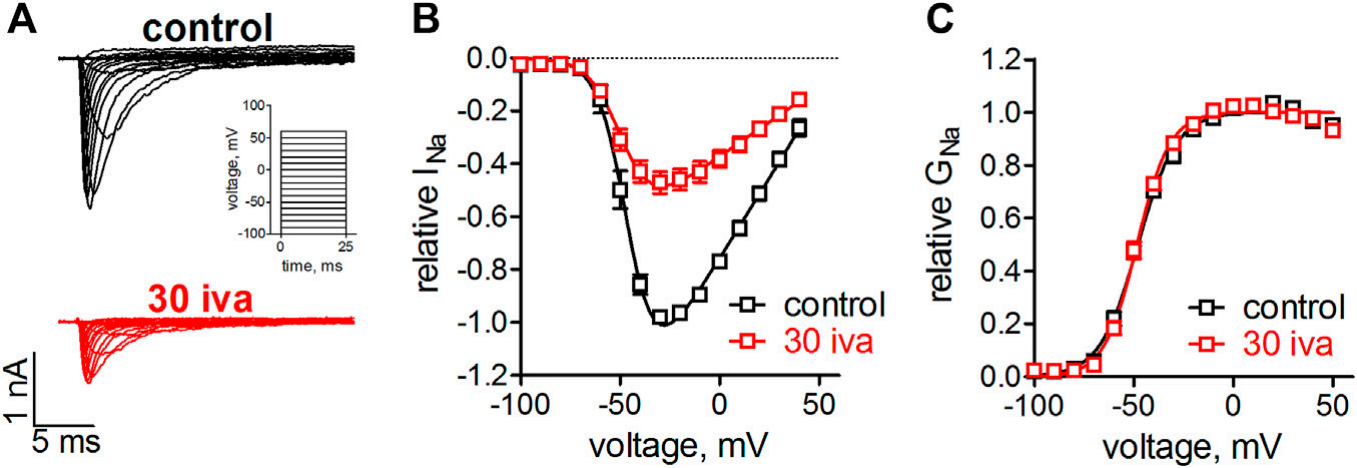
Effect of ivabradine on the voltage-dependence on activation in human Na_v_1.5 channels. tsA-201 cells expressing human Na_v_1.5 channels were held at a holding potential of −100 mV and sodium currents were elicited by 25 ms, rectangular steps to various voltages (in 10 mV increment, see inset in **A**). **(A)** Original current traces as elicited by the voltage-clamp protocol in the absence (top, black) and presence of 30 µM ivabradine (bottom, red). **(B)** Current-voltage relationships as derived from the maximal inward current at the applied voltages. **(C)** Conductance-voltage relationships as derived from **B**. No significant differences were found between the half point of voltage-dependent activation, V_1/2_ = −48 ± 1 mV and −48 ± 1 mV (*p* = 0.7), and the steepness of activation, k = 9.7 ± 0.9 and 8.1 ± 0.6 (*p* = 0.15), for control (*n* = 5) and in the presence of ivabradine (*n* = 9), respectively.

We have previously shown that ivabradine inhibits Na_v_1.5 channels with an IC_50_ of 30 µM (Haechl et al., 2019). Voltage-gated ion channel inhibition by small molecules typically goes along with altered channel gating, i.e., changes in the voltage- and time-dependence of channel activation and inactivation. We first tested the effect of ivabradine on Na_v_1.5 channel activation. To this end, human Na_v_1.5 channels were heterologously expressed in tsA-201 cells and sodium currents through these channels were elicited from a holding potential of −100 mV by depolarizing, rectangular voltage steps of 25 ms duration (Figure 1A). Inward current maxima were determined for every step and respective values were plotted as a function of the applied voltages to obtain current-voltage relationships (Figure 1B) and the voltage-dependence of Na_v_1.5 activation (Figure 1C). The same voltage-clamp protocol was repeated after 3 min equilibration with ivabradine. As can be seen from the original current recordings (Figure 1A), IV relationships (Figure 1B) and activation curves (Figure 1C), 30 µM ivabradine reduced sodium currents by 50% but did not alter the voltage-dependence of activation (Figure 1C).

### Ivabradine Does Not Affect Steady-State Inactivation of Na_v_1.5

**FIGURE 2:**
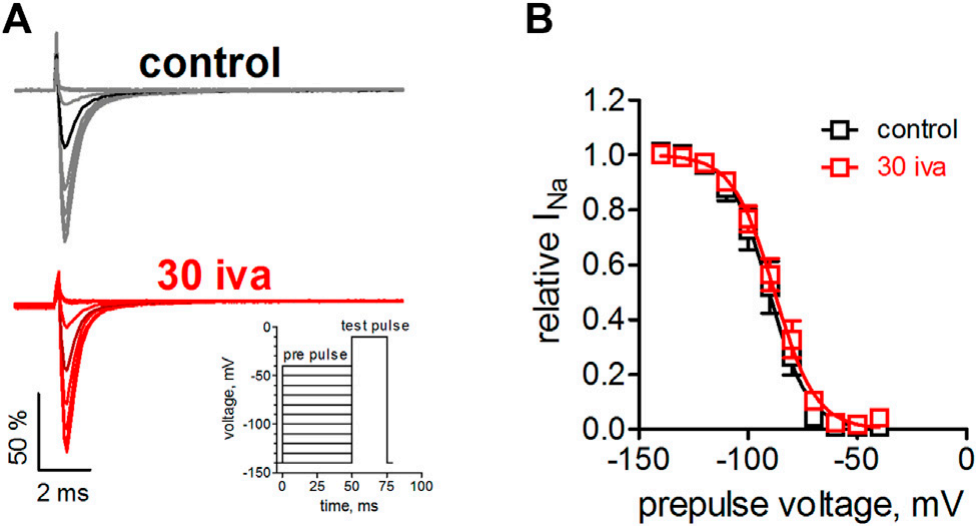
Effect of ivabradine on the steady-state fast inactivation in human Na_v_1.5 channels. **(A)** Voltage-clamp protocol and original current traces in the presence and absence of 30 µM ivabradine. tsA-201 cells expressing human Na_v_1.5 channels were held at a holding potential of −140 mV. An inactivating prepulse of 50 ms to various voltages preceded a 25 ms test pulse to −10 mV, to maximally activate the available channels (inset). Current traces for a prepulse to −90 mV are highlighted in a darker color. **(B)** Voltage-dependence of steady-state fast inactivation as derived from maximal inward current amplitude plotted against prepulse voltages. No significant difference was found between the half point, V_1/2_ = −90 ± 3 and −88 ± 2 (*p* = 0.6), and the steepness, k = 7.8 ± 0.3 and 8.7 ± 0.4 (*p* = 0.12), under control conditions (*n* = 6) and in the presence of ivabradine (*n* = 7), respectively.

We suspected that ivabradine, like many other agents targeting VGSCs, would alter Na_v_1.5 channel inactivation [e.g., (Lenkey et al., 2010)]. We, therefore, tested the effect of ivabradine on the voltage-dependence of steady-state fast inactivation. Inactivation was induced by a 50 ms inactivating prepulse to various voltages before testing the channel availability by a brief test pulse (Figure 2A inset). Ivabradine did neither alter the half point nor the steepness of the voltage-dependence of inactivation (Figure 2B).

**FIGURE 3:**
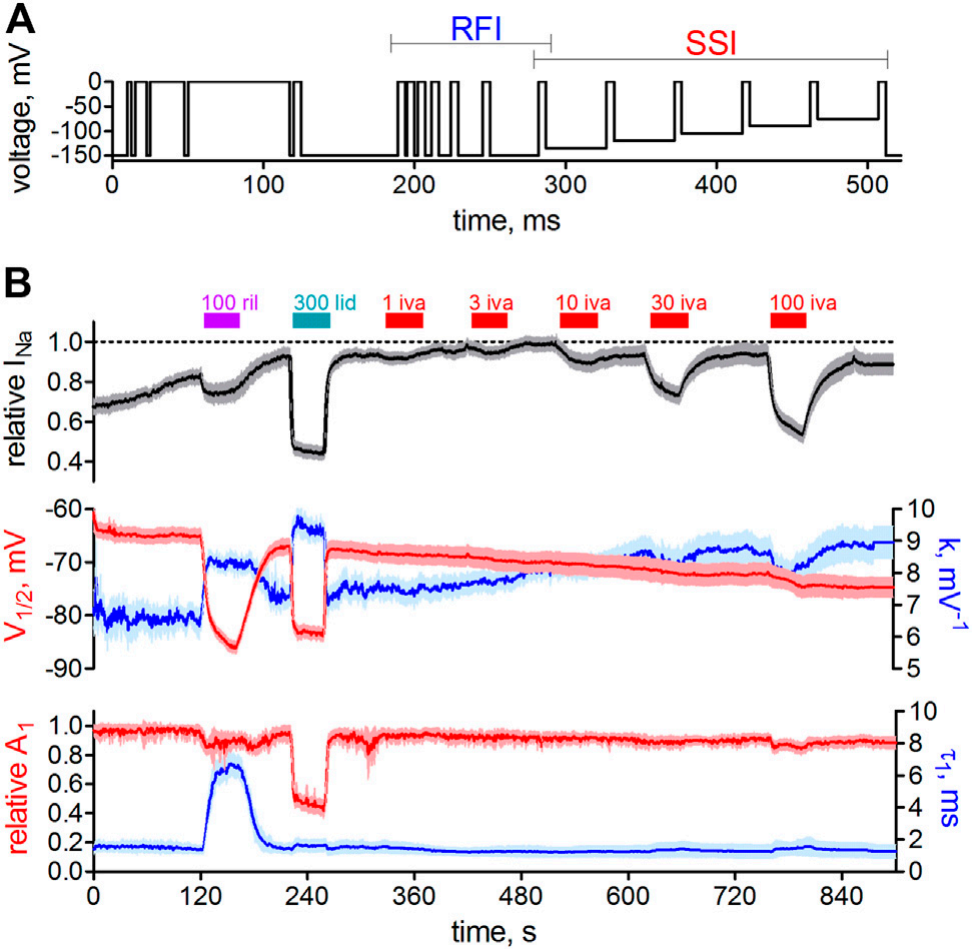
Automated patch-clamp analysis of the effect of ivabradine on human Na_v_1.5 fast inactivation. **(A)** Voltage-clamp protocol to simultaneously test for recovery from inactivation (RFI, blue bracket) and voltage-dependence of steady-state inactivation (SSI, red bracket) continuously once per second throughout the experiment. **(B)** Top: sodium current amplitude from hNa_v_1.5 channels stably expressed in HEK-293 cells. Mean current trace ± SD from *n* = 11 recording channels, with each channel recording the average of a maximum of 20 cells. Perfusion of different drugs is indicated by the above bars; 100 µM riluzole (100 ril), 300 µM lidocaine (300 lid), and increasing concentrations of ivabradine (1–100 μM; 1-100 iva). Middle: the parameters from automated fitting of the SSI plot at 1 s time resolution. Half point of voltage-dependent inactivation (V_1/2_; left axis, red) and steepness of respective voltage-dependence (k; right axis, blue). Bottom: Selected parameters from automated fitting of the RFI plot. Recovery was fit with a double-exponential function with the second, slow time constant constrained to 150 ms (see Methods). Relative amplitude of the fast time constant of recovery (A1; left axis, red) and respective fast time constant (τ1; right axis, blue). Time axis applies to all panels in **B**.

It is generally known that in whole-cell patch-clamp experiments voltage-dependence of Na_v_1.5 steady-state inactivation is prone to a time-dependent, hyperpolarizing shift (Wang et al., 1996). Under our experimental conditions, this shift presented linearly and amounted to a hyperpolarization of V_1/2_ values by about 1 mV per min (not shown). We certainly accounted for this shift when assessing drug effects on activation (Figure 1) and inactivation (Figure 2). Thus, we did not compare the measurements in the presence of ivabradine with those from preceding controls (before drug application; in the very same cell) but performed independent control measurements that followed the same time course. Although correctly accounting for the time-dependent shifts, this approach prevented an in-cell control at the same time. To further investigate the effects of ivabradine on Na_v_1.5 inactivation, we, therefore, employed a complementary approach relying on an automated patch-clamp platform. We used a newly developed voltage-clamp protocol that was optimized for high information content and high temporal resolution (see Methods for details). Briefly, it consisted of 17 depolarizing voltage steeps (Figure 3A), which were repeated every second throughout the time course of the experiment, and which allowed us to continuously assess steady-state inactivation (SSI) and recovery from inactivation (RFI). The holding potential was set to −150 mV to minimize voltage-dependent rundown of the current and to ensure that all channels are in the resting state.

To test the functionality of our approach, we first applied the well-characterized VGSC blockers riluzole (Földi et al., 2021) and lidocaine (Gawali et al., 2015). Application of these drugs led to a significant current reduction (Figure 3B, top panel) and a significant hyperpolarizing shift in the half point of inactivation, V_1/2_ (Figure 3B, middle panel, red trace), in accordance with named reports. Ivabradine, on the other hand, inhibited Na_v_1.5 channels in a concentration-dependent manner (Figure 3B, top panel) but did not shift the voltage-dependence of steady-state inactivation (Figure 3B, middel panel, red trace, right-hand side), consistent with the results from our manual patch-clamp experiments (Figure 2). The design of the employed voltage-clamp protocol also allowed us to assess the recovery from inactivation (Figure 3B, lower panel) at the same time. Riluzole and lidocaine prolonged the recovery from inactivation by slowing the fast time constant (τ_1_; Figure 3B, lower panel, blue, right axis), or by increasing the contribution of the slow time constant a decrease the relative amplitude of the fast time constant (A_1_; Figure 3B, lower panel, red, left axis), respectively. In contrast, the application of ivabradine did not alter recovery from inactivation at concentrations up to 30 µM (Figure 3B). Only at 100 µM did ivabradine appear to induce a small effect; however, we did not consider these changes to be of any relevance at therapeutic concentrations, and hence did not investigate it further. Taken together, our manual and automated patch-clamp results showed that ivabradine did not affect Na_v_1.5 fast inactivation.

### Inhibition of Na_v_1.5 Channels by Ivabradine Is Voltage-dependent

**FIGURE 4:**
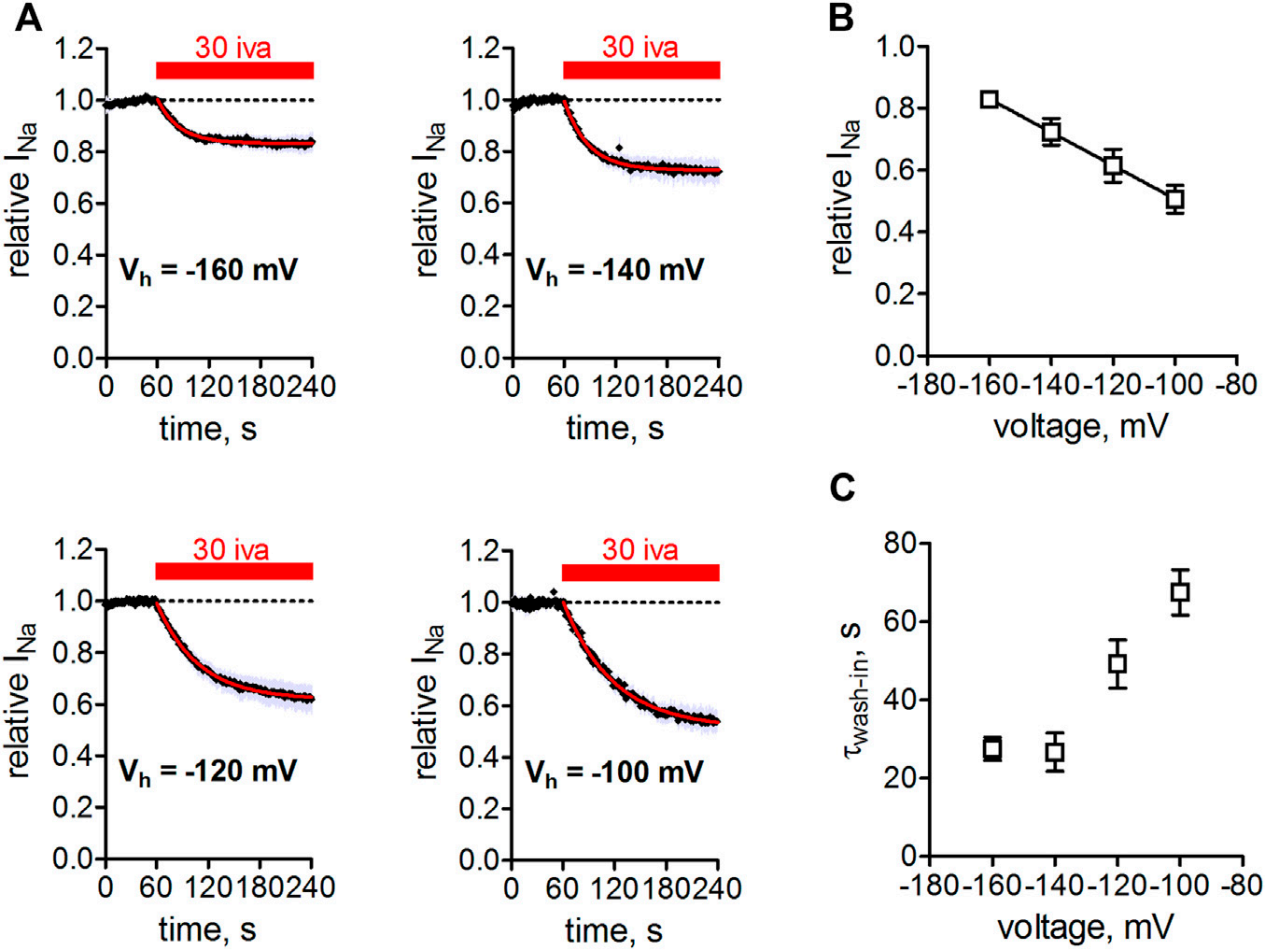
Voltage-dependence of Na_v_1.5 inhibition by ivabradine. **(A)** tsA-201 cells expressing human Na_v_1.5 channels were held at different holding potentials (V_h_ = −160 to −100 mV) and depolarized to -10 mV for 25 ms every second. After 60 s, 30 µM ivabradine was applied for 3 min (red bar). Current inhibition was fitted with a mono-exponential function to derive steady-state inhibition levels. Measurements at V_h_ = −100 mV were corrected for Na_v_1.5 current rundown caused by the mentioned time-dependent shift of inactivation in whole-cell experiments. **(B)** Summary of Na_v_1.5 current amplitude in the presence of 30 µM normalized to respective control before drug application and plotted over the applied V_h_. Data for V_h_ of −160, −140, −120, −100 mV are from *n* = 5, 5, 5, 5 independent measurements. **(C)** Summary of time constants as derived from single exponential fits to the wash-in of 30 µM ivabradine in **(A)**. All the values are given as mean ± SEM.

We next tested the action of ivabradine at different holding potentials (Figure 4). Application of ivabradine reduced Na_v_1.5 currents by 50% at a holding potential of -100 mV (Figure 4A bottom right), in full agreement with previous results (Figure 1, Haechl et al., 2019), but at a holding potential of −160 mV current reduction amounted to less than 20% (Figure 4A top left). Evaluating the current reduction for different holding potentials resulted in a linear relationship of Na_v_1.5 current inhibition (Figure 4B). Interestingly, current inhibition developed faster at hyperpolarized when compared to more depolarized holding potentials (Figure 4C).

### Inhibition of Na_v_1.5 Channels by Ivabradine Occurs in a Frequency-dependent Manner

**FIGURE 5:**
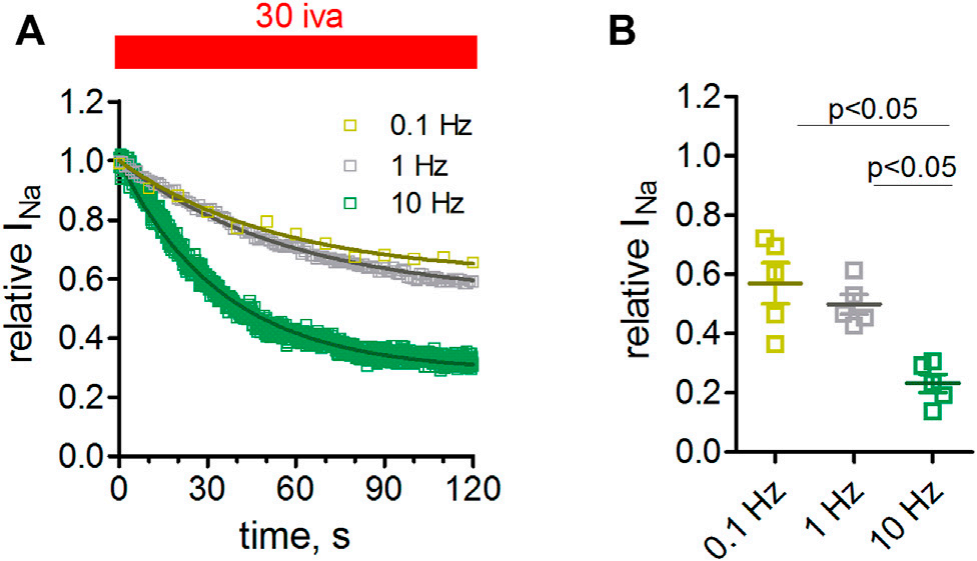
Frequency dependence of Na_v_1.5 inhibition by ivabradine. tsA-201 cells expressing human Na_v_1.5 channels were held at a holding potential of −100 mV and depolarized to -10 mV for 25 ms at a frequency of either 0.1, 1, or 10 Hz. **(A)** Maximal peak inward sodium current amplitude during −10 mV depolarizations was monitored over time. After stable amplitude had been established 30 µM of ivabradine was washed in. Current amplitude values in the continuous presence of 30 µM ivabradine were normalized to respective control values before drug application (norm Na_v_1.5 current). The level of inhibition was significantly lower at 10 Hz compared to 0.1 and 1 Hz pulse frequency. **(B)** Summary of inhibition levels as derived from single-exponential fits to the data shown in **A**; mean ± SEM from *n* = 5, 5, and 5 independent experiments for 0.1, 1, and 10 Hz, respectively.

We next tested the effect of ivabradine at different pulsing frequencies. To this end, we held the cells at -100 mV and depolarized them to −10 mV for 25 ms at frequencies of 0.1, 1, and 10 Hz. Higher frequencies led to a stronger channel inhibition (Figures 5A,B). This effect was significant when comparing a pulsing frequency of 10 with 1, or 0.1 Hz.

### Inhibition of Na_v_1.5 Channels by Ivabradine Is Temperature- and Use-dependent

**FIGURE 6:**
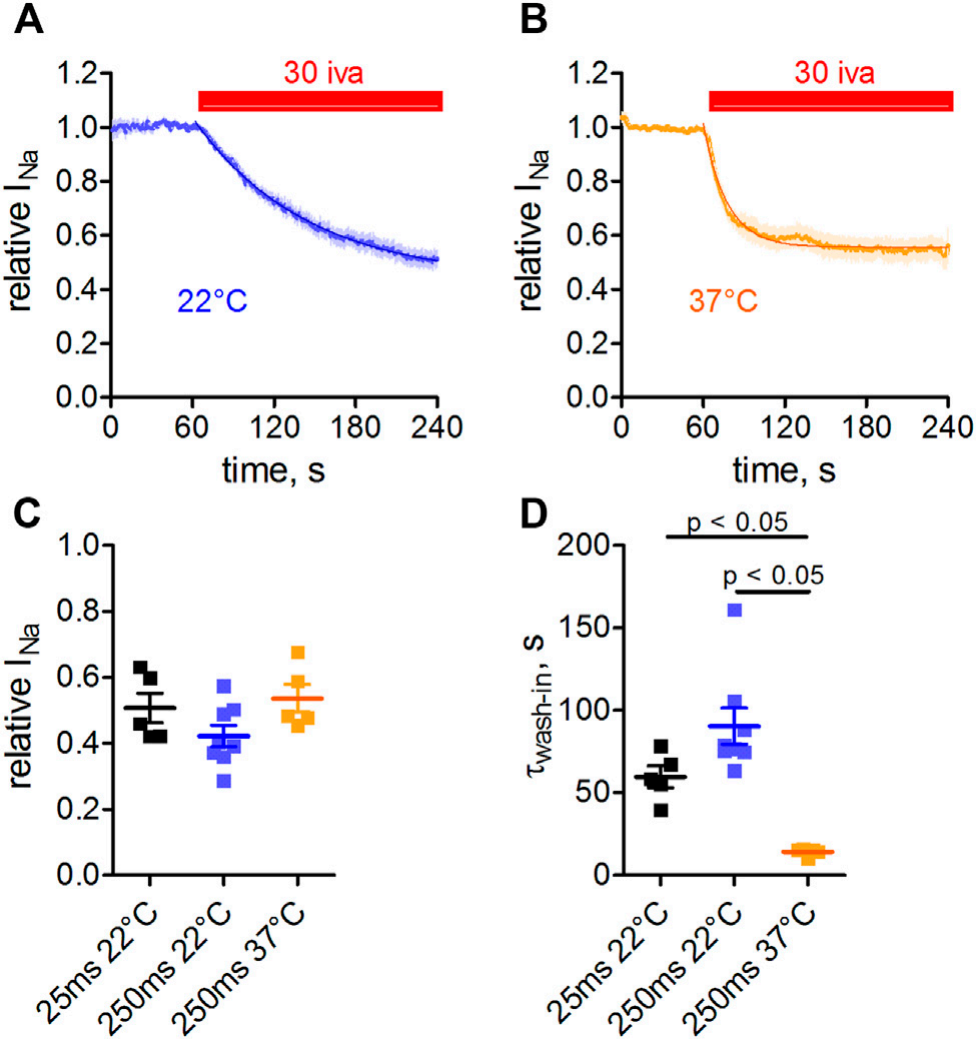
Temperature- and use-dependence of hNa_v_1.5 inhibition by ivabradine. tsA-201 cells expressing human Na_v_1.5 channels were held at a holding potential of −100 mV and sodium currents were elicited by depolarizing voltage steps to −10 mV for 250 ms every second. Normalized maximal Na_v_1.5 current amplitude in the absence (first 60 s) and presence of 30 µM ivabradine (3 min application, as indicated) at a temperature of either 22°C **(A)** or 37°C **(B)**. **(C)** Na_v_1.5 current inhibition at different depolarization times and temperatures. Values for depolarization times of 25 and 250 ms are plotted in the 1^st^ and 2^nd^ column, respectively (values for 25 ms depolarization are replotted from Figure 5). Values for temperatures of 22 and 37°C with depolarization for 250 ms are plotted in the 2nd and 3rd columns, respectively. **(D)** As in **C**, but for the wash-in time constants. Data are expressed as mean ± SEM (*n* = 5, 8, 5).

Measurements were performed at room temperature throughout this study. To check whether the observed affinity of ivabradine toward Na_v_1.5 would markedly change at physiological temperatures, we performed measurements at 37°C (Figure 6). In these experiments, we also increased the pulse width of depolarization from 25 to 250 ms in order to test for a use-dependent inhibition and to better mimic the shape of the human cardiac action potential. Inhibition of Na_v_1.5 currents developed at a slower rate at room temperature (Figures 6A,D) than at 37°C (Figures 6B,D); therefore, steady-state levels were reached sooner at 37°C. The amount of inhibition at 37°C was comparable to that at 22°C (Figures 6C, 2nd, and 3rd column), as was the inhibition for the different depolarization lengths tested; comparable block levels for 25 and 250 ms depolarisation (Figure 6C, 1st, and 2nd column).

### Ivabradine Inhibits Different VGSC Isoforms

**FIGURE 7:**
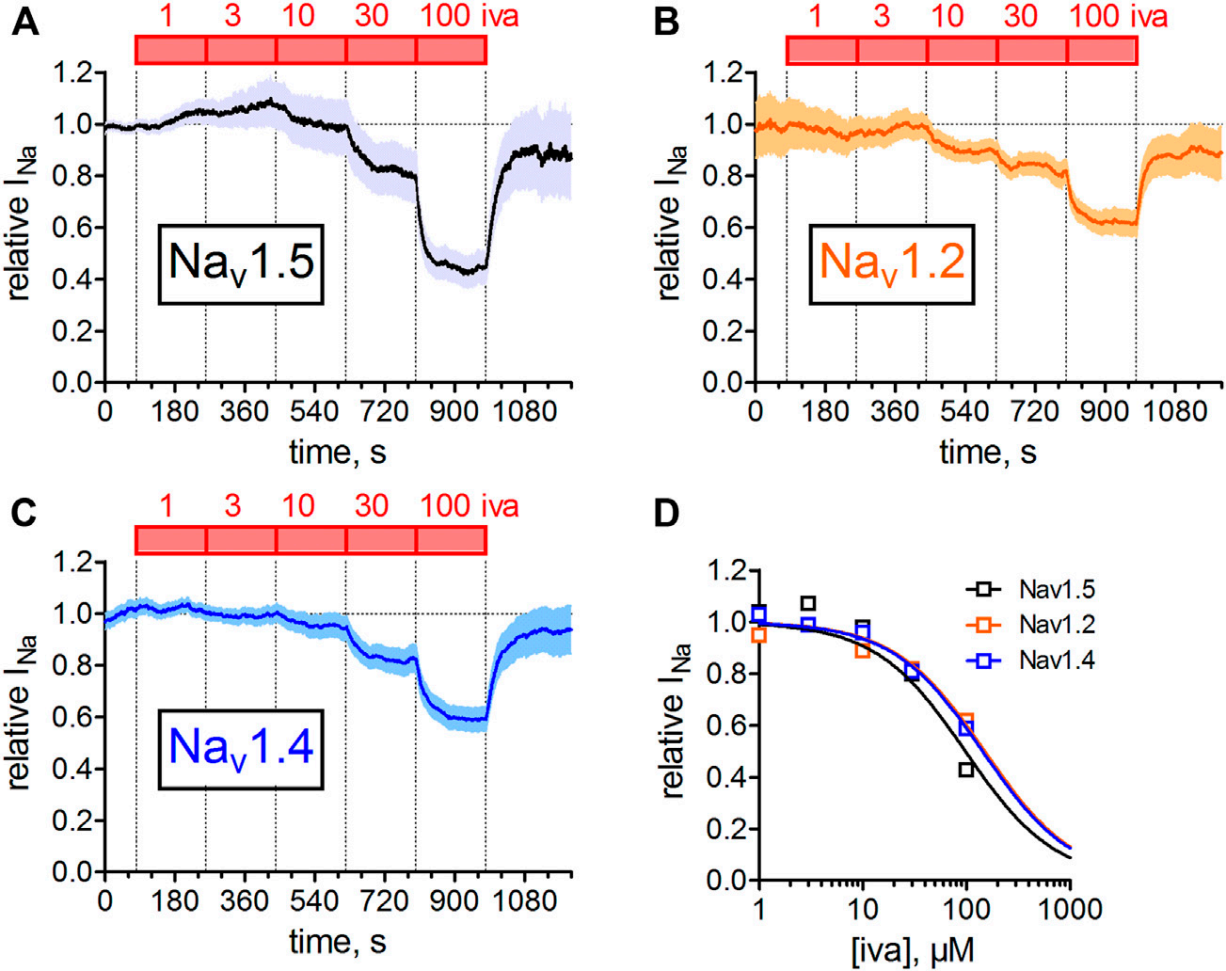
Concentration-dependent inhibition of different VGSC isoforms. Automated patch-clamp recordings of sodium currents through Na_v_1.2, Na_v_1.4, and Na_v_1.5 channels. Sodium currents were elicited from a holding potential of −150 mV by 25 ms depolarizing voltage steps to −10 mV. Ivabradine was applied at increasing concentrations (1, 3, 10, 30, 100 µM) for 3 min each. Mean data from 12, 15, and 12 channels are shown for Na_v_1.2, Na_v_1.4, and Na_v_1.5, respectively. Data were fit to a Hill equation with a Hill coefficient of n_H_ = 1 to estimate half points of inhibition. IC_50_ values (mean ± SEM) amounted to 296 ± 11, 257 ± 15, and 137 ± 8 µM for Na_v_1.2, Na_v_1.4, and Na_v_1.5. One-way ANOVA with Tukey’s post-hoc test revealed a statistical difference between Na_v_1.5 and Na_v_1.2 (*p* < 0.001) and Na_v_1.4 (*p* < 0.001), but not between Na_v_1.2 and Na_v_1.4.

Na_v_1.5 is the predominant isoform expressed in the mammalian heart (Zimmer et al., 2014); smaller contributions stem from neuronal or the skeletal muscle channel isoform (Haufe et al., 2007; Zimmer et al., 2014). In particular, neuronal VGSC isoforms have been suggested to play an important role in SAN automaticity (Haufe et al., 2007). We, therefore, compared the inhibition of ivabradine on Na_v_1.5 with its inhibition on one representative of the neuronal channel isoforms (Na_v_1.2) and with the skeletal muscle channel isoform (Na_v_1.4). Figure 7 shows the concentration-response curves for the three tested isoforms. Note that in these experiments the holding voltage was −150 mV, and hence current inhibition was not as pronounced as compared to a holding of −100 mV, in accordance with the voltage-dependence of Na_v_1.5 inhibition by 30 µM ivabradine (Figure 4).

### Ivabradine Inhibits Native VGSCs in Primary Cardiomyocytes

**FIGURE 8:**
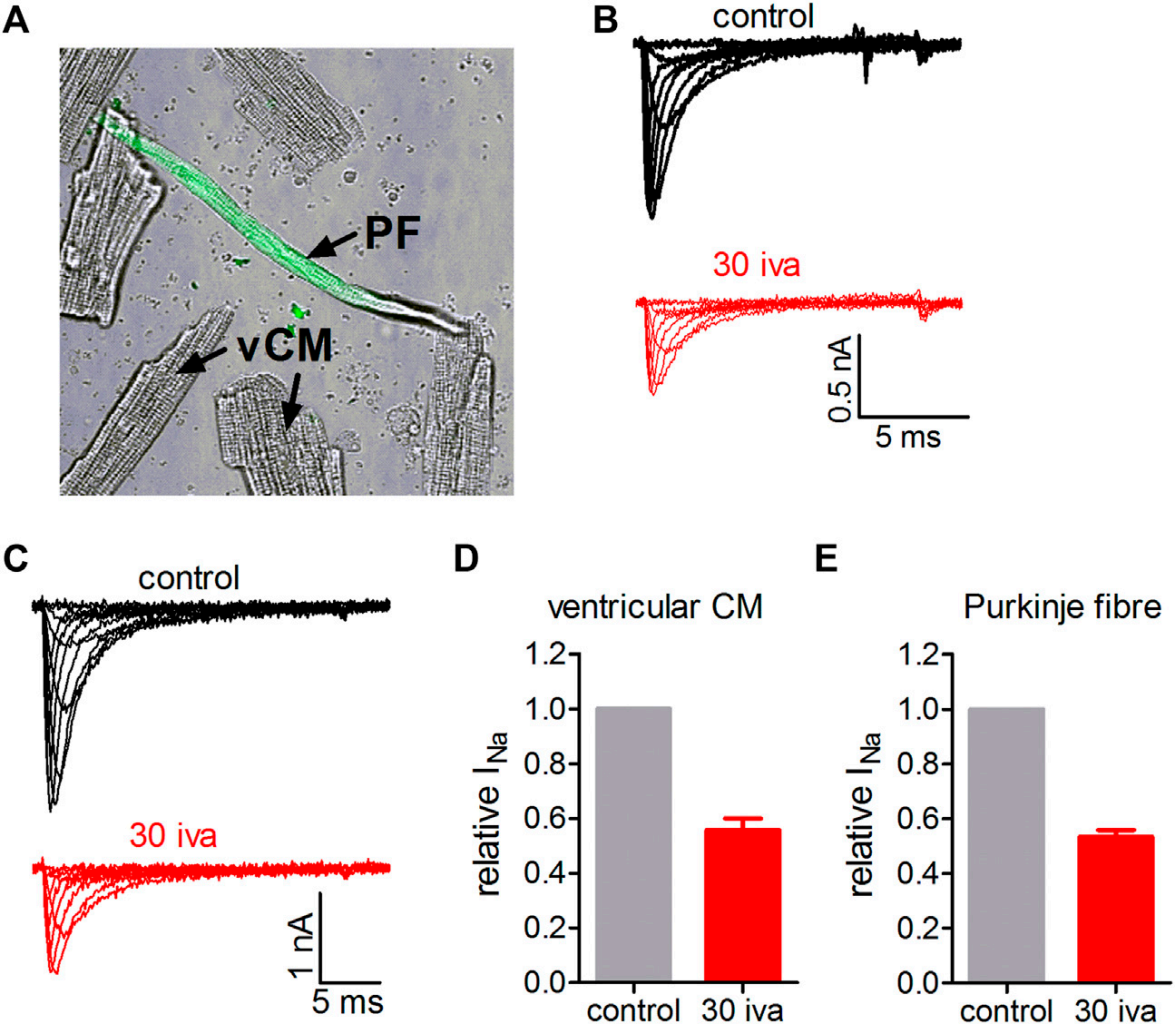
Effect of ivabradine on VGSCs in primary cardiomyocytes isolated from the ventricles and the conduction system of the mouse heart. **(A)** Transmitted light (TM) image of a typical ventricular cardiomyocyte (vCM) and a cardiomyocyte isolated from the conduction system of the heart (Connexin40-eGFP positive Purkinje fiber (PF); the green fluorescent light channel is overlaid with TM channel). **(B)** Tyoical original sodium current traces in a ventricular cardiomyocyte elicited from a holding potential of -70 mV by various voltage steps between −60 and +20 mV. Sodium currents under control conditions and in the presence of 30 µM ivabradine (30 iva; in red). **(C)** As in **B** but for Connexin40-eGFP positive cardiomyocytes isolated from the cardiac conduction system. **(D)** Summary of fractional sodium current levels before (control) and after equilibration with 30 µM ivabradine derived from *n* = 7 ventricular cardiomyocytes. **(E)** Summary of fractional sodium current levels before (control) and after equilibration with 30 µM ivabradine derived from *n* = 7 Purkinje fibers. Data are given as mean ± SEM.

Next, we wanted to know if ivabradine would also inhibit native Na_v_1.5 channels in primary cardiomyocytes. To this end, we isolated murine cardiomyocytes using a Langendorff heart preparation. We first tested the effect of ivabradine on ventricular cardiomyocytes. Figure 8 shows that 30 µM ivabradine inhibited VGSCs in these cells by about 50% (Figures 8B,D) comparable to the observed IC_50_ value on heterologously expressed Na_v_1.5 channels (Figure 1, Haechl et al., 2019). Second, we wanted to know if this would also hold true for VGSCs in the conduction system of the heart. To this end, we isolated cardiomyocytes from a knock-in mouse line expressing GFP under the control of the *Cx40* gene. The corresponding protein, connexin-40, is strongly expressed in the cardiac conduction system but is absent in ventricular cardiomyocytes (Miquerol et al., 2004). The expression of eGFP allowed us to specifically select Purkinje fibers (PF) within all other cardiomyocytes as obtained from the Langendorff isolation (Figure 8A; Methods). These cells showed increased sodium current densities as compared to ventricular cells (compare representative current amplitudes in Figures 8B,C), in line with previous reports. The application of 30 µM ivabradine inhibited sodium currents in PF by about 50% (Figure 8E).

### Potential Molecular Interaction of Ivabradine With Na_v_1.5

**FIGURE 9:**
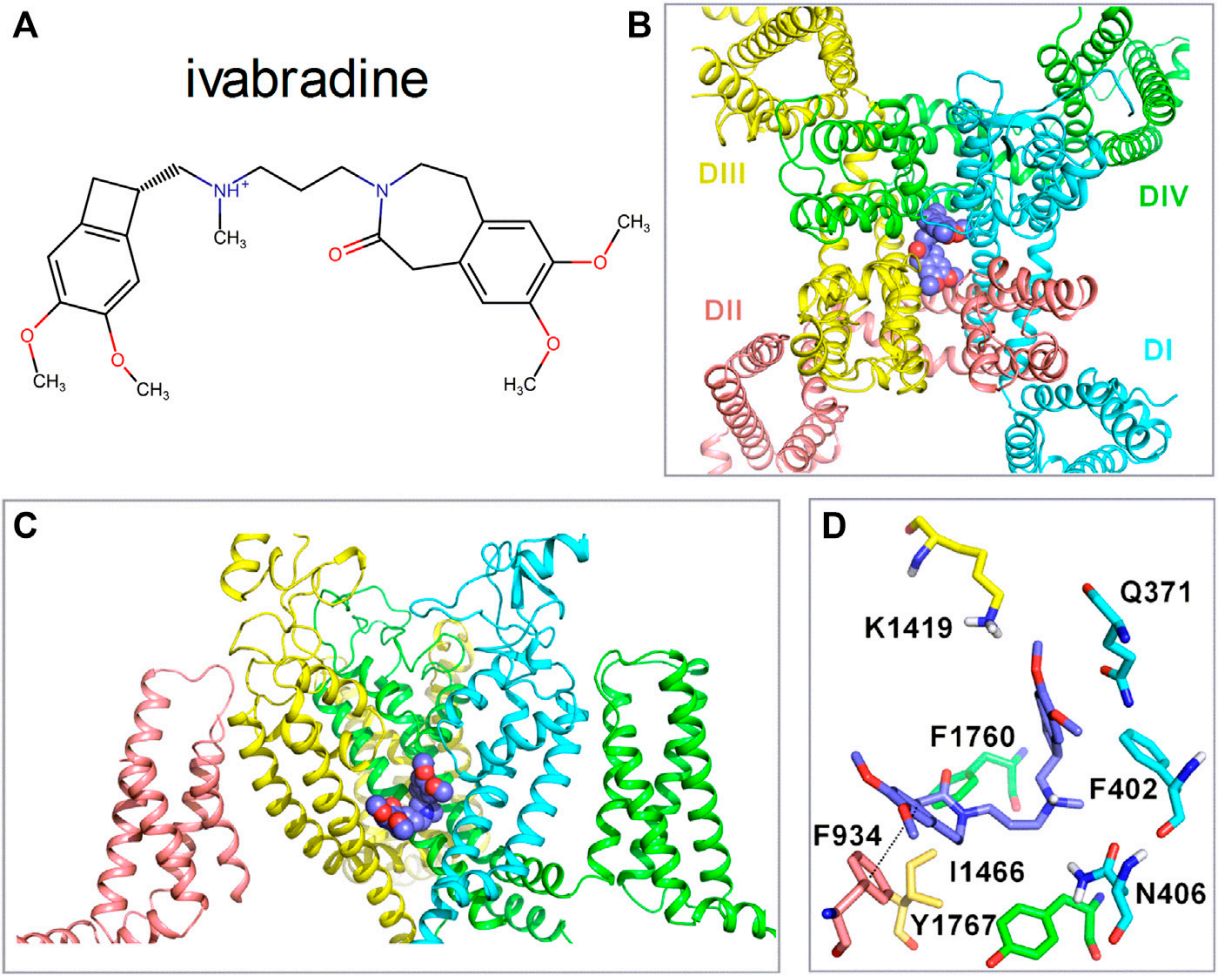
Molecular interactions of ivabradine with Na_v_1.5. **(A)** Chemical structure of ivabradine as used for the drug docking with a charged amine nitrogen at physiological pH. **(B)** Representative docking pose of Ivabradine shown as purple spheres in the cavity of Na_v_1.5 (cartoon representation) viewed from the top. **(C)** Side view of the binding site below the selectivity filter, with the voltage sensor of domain II hidden for clarity. **(D)** Residues within 5 Å of ivabradine are shown in stick representation. Dotted lines between F934 and Ivabradine denote π- π interactions. The distance between the two benzene rings is 4.1 Å.

Up to this point, we examined the biophysical properties of ivabradine. Here, we provide additional information regarding the potential molecular basis of the interaction of ivabradine with Na_v_1.5. Previously, ivabradine has been suggested to bind in the internal cavity of HCN4 channels, similar to the proposed binding mode of local anesthetics in VGSCs channels (EMA-Europe, 2005). Hence, we examined whether the cavity of Na_v_1.5 channels would provide interacting residues for the binding of ivabradine. Molecular drug docking in Figure 9 suggests that ivabradine binds to the central cavity of Na_v_1.5 in a kinked conformation, where it mainly forms hydrophobic and aromatic interactions with the channel. Similar to flecainide (Jiang et al., 2020), ivabradine binds below the selectivity filter by physically blocking sodium ion flux. Π-π interactions between the benzazepine moiety and F934 from domain II are predicted. The protonated amine does not form cation- π interactions in agreement with a previous study, suggesting that drugs with elongated linkers display kinked backbone conformations, precluding such cation-pi interactions (Pless et al., 2011).

## Discussion

### Ivabradine Is an Atypical Blocker of VGSCs

Ivabradine acts as an atypical inhibitor of VGSCs. Most blockers of VGSCs bind to the inactivated protein conformation(s) and stabilize the respective channel states by forming an energetically favorable drug-receptor complex. Thus, typically, entry into inactivation is accelerated and recovery from inactivation is delayed upon binding, while the voltage-dependence of inactivation is shifted to more hyperpolarized voltages. This, however, was not observed for ivabradine as the respective parameters were unaltered in the presence of the drug (Figures 1–3). To the best of our knowledge, this indifference to channel states is a novel phenomenon of VGSC inhibition. This is particularly astounding as our data are consistent with ivabradine binding to the classical local anesthetic binding site (Figure 9).

The onset of current inhibition was relatively slow (Figure 5A) as the block by ivabradine developed over a period of several minutes. Such slow development of block may result from binding to a slowly developing inactivated state. Alternatively, the drug may bind to fast inactivated states, albeit in a very slow fashion. Given the slow onset of block development it is very difficult to distinguish between these two possible modes of action and further studies will be needed to clarify this point. On the other hand, the block was not accelerated with concentration as the law of mass action would predict. This suggests a rate-limiting step along the access pathway may be related to the charged amine nitrogen or the specific chemical structure of ivabradine. The onset kinetics of hERG (Perissinotti et al., 2019) and hHCN4 (Bucchi et al., 2013) channel block by ivabradine were on a comparable timescale when accounting for the difference in respective experimental temperature in the latter. This suggests that onset may not reflect association itself, but more likely the process of drug diffusion and/or partitioning, which may be a common denominator of HCN, hERG, and Na_v_1.5 channel block.

In the manual patch-clamp experiments, we used 50 ms pre-pulses to test for steady-state fast-inactivation. This might not be long enough for certain compounds to associate, but the complex voltage-clamp protocol that we used in the automated patch experiments (shown in Figure 3) a series of pre-pulse (pulses #12 to #17) gives prolonged depolarization during which there was no return to the holding potential for a total of 230 ms. The advantages of this cumulative arrangement over the conventional SSI protocol are discussed in Lukacs et al. (2021). The fact that there was no detectable change in the half inactivation voltage up to 30 µM (Figure 3) indicates that ivabradine did not noticeably affect the resting-inactivated equilibrium also at these depolarization times. In addition, even longer depolarization (to −10 mV for up to 600 ms) did not increase the amount of inhibition observed with 30 µM ivabradine (data not shown).

Taken together, there is the possibility that the association of ivabradine is too slow to be seen with the used pre-pulse times in our experiments testing for fast inactivation. Nevertheless, the “pharmacological fingerprint” of ivabradine regarding VGSCs is atypical in the sense that it does not induce any kinetic changes within the time framework that is relevant to cardiac action potential durations, i.e., up to several hundred milliseconds.

### Ivabradine Plasma Concentrations and Physiological Relevance

The maximal free plasma concentration of ivabradine upon standard dosing (5 mg bid) was found to be 22 ng/ml, or equivalently about 50 nM (EMA-Europe, 2005). Even at high dosage, maximal free plasma concentrations of 100 nM are more than one order of magnitude lower than the reported IC_50_ value for the inhibition of HCN [2 μM; (Bucchi et al., 2006)], hERG [2–11 μM; (Melgari et al., 2015; Haechl et al., 2019)] and Na_v_1.5 channels [30 μM; (Haechl et al., 2019)]. Why should any of these interactions and in particular Na_v_1.5 inhibition with the lowest observed affinity among those, play a physiological role? Now, it is generally accepted that ivabradine reduces the heart rate by inhibiting HCN channels. Also, the drug carries a reported risk for QT-interval prolongation most commonly caused by hERG potassium channel inhibition. The fact that ivabradine exerts a pharmacological effect, despite the affinities for both HCN and hERG channels being substantially lower than the reported plasma concentrations, suggests that prevalent tissue concentrations must be significantly higher. Most likely this occurs by an accumulation of the lipophilic drug in lipid membrane compartments embedding respective ion channel proteins. Regarding Na_v_1.5, Koncz et al. observed a reduction in AP amplitude and upstroke velocity in dog Purkinje fibers - a physiological effect intimately linked to the inhibition of Na_v_1.5 channels-already at concentrations as low as 1 µM (Koncz et al., 2011), and hence within a concentration range well below the reported IC_50_ value of HCN. Moreover, in Amstetter et al., we recently reported that 5 min after i.p. administration of 10 mg/kg ivabradine to anesthetized mice the spontaneous heart rate had declined by ∼13%, which is within the range observed in human clinical studies. At the same time, a significant increase in QRS duration by ∼18% was observed, suggesting a reduction in the ventricular conduction velocity, presumably by the block of VGSCs (Amstetter et al., 2021). This suggests that plasma levels of ivabradine associated with moderate reductions in heart rate may be associated with inhibition of VGSCs.

### Pro- and Anti-arrhythmic Potential of Ivabradine

Administration of ivabradine has proven safe and without pro-arrhythmic prevalence in clinical trials (Savelieva and Camm, 2008; Borer and Tardif, 2010), a pro-arrhythmic potential might thus emerge only under specific pathological conditions (Koncz et al., 2011). On the other hand, accumulating evidence points toward a promising anti-arrhythmic potential of the drug (see Introduction). Three potential mechanisms are worth to be considered here.

First, VGSCs contribute to controlling sinus rhythm in the SAN. Despite almost absent mRNA and protein levels in the SAN core region, VGSCs are robustly expressed in the SAN periphery where they substantially contribute to impulse conduction (Milanesi et al., 2015). A growing list of genetic variants within the SCN5A gene induce significant dysfunction of the SAN and the cardiac conduction system and are associated with bradycardia and sinus-exit block (Benson et al., 2003; Lei et al., 2005). Moreover, in intact mammalian SAN preparations, VGSC block was shown to reduce the threshold of diastolic depolarization (Baruscotti et al., 1996; Muramatsu et al., 1996; Létienne et al., 2006), and dose-dependently reduced the heart rate with (Abraham et al., 1989) and without (Gilmour et al., 1984) autonomous regulation. VGSC block was also associated with conduction failure and re-entrant arrhythmias in the human SAN (Li et al., 2020; Vik-Mo et al., 1982; Kim et al., 2011; LaBarre et al., 1979). The observed inhibition of VGSCs across channel isoforms (Figure 7) and in native cardiomyocytes (Figure 8), the important role of VGSCs in SAN function, and the overlapping concentration range with HCN4 channel inhibition (Bucchi et al., 2006; Thollon et al., 2007), therefore suggests a contribution of VGSC inhibition to the bradycardic action and the control of sinus tachycardia associated with administration of ivabradine.

Second, VGSCs are expressed in the human AV node (Greener et al., 2011) and loss-of-function mutations in respective VGSC genes are associated with delayed AV-node conduction (Milanesi et al., 2015; Papadatos et al., 2002). Moreover, VGSC blockers like lidocaine and flecainide prolong AV nodal conduction times and have been reported to induce AV-block (Vik-Mo et al., 1982; Lieberman et al., 1968; Estes et al., 1984; Hellestrand et al., 2007; Lichstein et al., 1973; Grenadier et al., 1982). In line with the inhibition of VGSCs, ivabradine prolonged AV-nodal conduction in guinea pigs (Verrier et al., 2014) and mice (Amstetter et al., 2021), and zatebradine, a precursor of ivabradine, induced a prolongation of the atrial-His interval in a canine model of disrupted SA function (Yamazaki et al., 1995). Blockade of HCN4 channels expressed in the AV-node has been put forward as a respective mechanism (Verrier et al., 2014), but inhibition of VGSCs offers an alternative/additional interpretation. Thus, inhibition of VGSC by ivabradine may at least in part explain the potential rate-controlling properties of the drug. Worth noting in this context is that the block of Na_v_1.5 by ivabradine was increased upon depolarized potentials (Figure 4); as such it would block VGSCs more effectively in atrial cells and cells from the cardiac Purkinje system, in which resting potentials are significantly more depolarized as compared to the ventricular cells (Pandit, 2014). This effect, however, was masked when we compared VGSC block in ventricular and Purkinje cells as shown in Figure 8, because both cell types were voltage-clamped to the same holding potential.

Third, most but not all VGSC blockers are associated with a prolongation of the QRS interval of the surface ECG (Harmer et al., 2011); this was not observed for ivabradine in small human cohort studies (Camm and Lau, 2003; De Ferrari et al., 2008). However, the European Medical Association (EMA) acknowledged respective changes in its official report on ivabradine (“The changes noted in the PR interval and the QRS duration with ivabradine were of no clinical concern” (EMA-Europe, 2005)). In animal models, a change in QRS duration was observed (Amstetter et al., 2021) but not by others (Milliez et al., 2009; Leoni et al., 2005). Respective His-Ventricle (HV) intervals show a similar picture; a trend toward an HV prolongation in several small clinical studies (Savelieva and Camm, 2006), and a trend toward HV interval prolongation in guinea pigs (Verrier et al., 2014). Overall it seems that ivabradine affects VGSCs in the ventricles at clinically relevant dosage, but only to a small extent. A reason might be provided by the frequency dependence of the channel block (Figure 5). As such, inhibition of VGSCs channels is weaker at low heart rates, which is further promoted by the drug, while the block becomes stronger when heart rates increase. It is therefore likely that ivabradine only minimally affects HV and QRS interval times at resting heart rates, under which routine ECGs are recorded, but that its effect gradually increases upon tachycardia. However, the observed frequency dependence for ivabradine was weak. Clinically used VGSC blockers, in particular class Ic antiarrhythmics, typically exhibit a strong preference for the open/inactive VGSC states resulting in a pronounced frequency-dependent block and a rate-dependent slowing of ventricular conduction. A weak frequency dependence may also serve as an explanation that changes in QRS duration by ivabradine were not rate-dependent (Amstetter et al., 2021). In any case, ivabradine was recently shown to control catecholaminergic polymorphic ventricular tachycardia (Vaksmann and Klug, 2018; Kohli et al., 2020) and junctional ectopic tachycardia (Al-Ghamdi et al., 2013; Dieks et al., 2016; Kumar et al., 2017; Ergul et al., 2018; Ergul and Ozturk, 2018; Mert et al., 2018; Janson et al., 2019; Kumar et al., 2019). While some of these arrhythmias may be of automatic origin, potentially tied to the function of HCN channels, the antiarrhythmic activity of ivabradine could also stem from the inhibition of VGSCs, in particular under ischemic conditions and in the failing heart, when resting membrane potentials are substantially depolarized (Bean et al., 1983) and channel block would be favored (Figure 4).

### Further Clinical Relevance

Beyond a well-established bradycardic and potential anti-arrhythmic action, recent studies suggest ivabradine possesses additional cardio-protective effects. Thus the drug has proved beneficial in patients after heart transplantation (Rivinius et al., 2020) and in the long-term treatment of post-infarct patients (Suffredini et al., 2012). Here, ivabradine could significantly reduce infarct size independent of the heart rate (Heusch, 2008; Kleinbongard et al., 2015). The fact that VGSC blockers have long been considered to be beneficial in reducing infarct size area (Nasser et al., 1980; Vitola et al., 1997; Kaczmarek et al., 2009), prompts us to speculate that the block of VGSCs by ivabradine contributes to this phenomenon.

Finally, the block of neuronal VGSC isoforms may contribute to the anticonvulsive effects of ivabradine (Cavalcante et al., 2019; Iacone et al., 2021). A plethora of gain-of-function mutations within the neuronal VGSC genes has been associated with different forms of epilepsy (Menezes et al., 2020). Thus, inhibition of VGSCs by ivabradine, e.g., of Na_v_1.2 predominantly expressed in principal neurons, could potentially lead to the depression of excess neuronal firing that spontaneously occurs in various forms of epilepsy. Considering that current inhibition was most pronounced at high-frequency discharge patterns (Figure 5), and at depolarized membrane potentials (Figure 4), the block of VGSCs by ivabradine may help to suppress epileptic seizures.

## Conclusion

Ivabradine is an atypical blocker of VGSCs with potential pro- and anti-arrhythmic properties. Block of VGSCs by ivabradine likely contributes to the lowering of heart rate and slowing of AV conduction observed upon administration of this drug.

## Data Availability

The original contributions presented in the study are included in the article/Supplementary Materials, further inquiries can be directed to the corresponding author.
