# Supplementary material for: The Bradycardic Agent Ivabradine Acts as an Atypical Inhibitor of Voltage-Gated Sodium Channels
Source: Front Pharmacol. 2022 May 2;13:809802. doi: 10.3389/fphar.2022.809802 (PMC9108390; doi:10.3389/fphar.2022.809802)

**Supplemental Material – Drug docking**

Standard parameters for drug docking were used as already described in the Methods. This can be seen in the provided drug docking configuration file gold.conf. Other provided files include the docking input (XXX_protein.mol2) and ligand files (flecainide.mol2 and ivabradine-drugbank.sdf) with respective information of origin given below.

<u>Drug docking configuration file:</u>

**gold.conf**

Configuration file including all parameters used for gold docking

Other provided files include the docking input and ligand files.

<u>Docking input file</u>:

**XXXX_protein.mol2**

The cryo-EM structure 6UZ3 of the cardiac sodium channel Nav1.5 (published by Jiang et al. 2020, Cell 180: 122-134.e10, DOI: 10.1016/j.cell.2019.11.041) served as input for gold docking. It was downloaded from the RCSB protein data bank (https://www.rcsb.org), PDB DOI: 10.2210/pdb6UZ3/pdb6UZ3.

<u>Ligand files</u>:

**flecainide.mol2**

The structure of flecainide was extracted from the cryo-EM structure 6UZ0 published by Jiang et al. 2020, Cell 180: 122-134.e10, DOI: 10.1016/j.cell.2019.11.041),

**ivabradine-drugbank.sdf**

The structure of ivabradine was downloaded from drugbank (https://go.drugbank.com/drugs) with DrugBank Accession Number DB09083

**Supplemental table 1 | Docking scores of the 20 best ranked ivabradine conformations**. Best ranked flecainide conformation is given for comparison.

| Docking solution (#) | Score |
|---|---|
| 37 | 89.8530 |
| 31 | 84.2303 |
| 42 | 82.1043 |
| 39 | 78.6278 |
| 29 | 77.6626 |
| 10 | 76.7844 |
| 26 | 76.1572 |
| 8 | 75.1424 |
| 12 | 75.0144 |
| 14 | 74.7998 |
| 38 | 74.2781 |
| 35 | 74.1914 |
| 43 | 74.0551 |
| 45 | 73.8702 |
| 5 | 73.3750 |
| 18 | 73.1614 |
| 1 | 73.0528 |
| 4 | 73.0246 |
| 3 | 72.9223 |
| 30 | 72.6395 |
| Flecainide | 77.5817 |

**Supplemental figure 1 | 19 highest ranked docking poses of ivabradine**. Except for 2 poses, all bound ivabradine molecules adopt a kinked conformation.

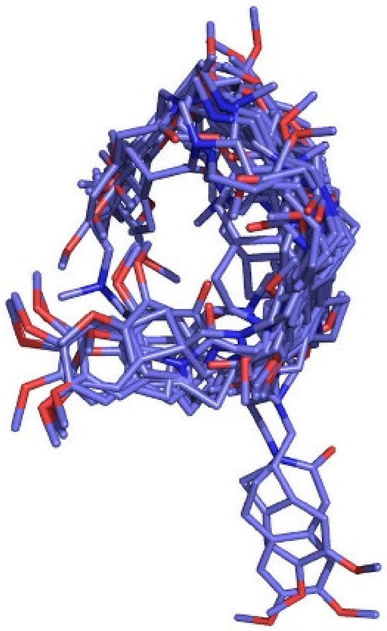

Supplement: Supplementary file 1 [file Presentation1.pdf]
